# Military Personals

**Published:** 1918-12

**Authors:** 


### MILITARY PERSONALS

Dr. Frederick W. Parsons of Poughkeepsie, a graduate of the University of Buffalo, has been in medical service in France for nearly two years. In August, he was placed in charge of Base Hospital 117 and, on Oct. 1, was promoted to his majority.

Capt. A. L. Benedict of Buffalo, was transferred from the Gas Defense Plant, Long Island City, to the Base Hospital, Camp Dix, Nov. 9.

Lt. Milton Bork of Buffalo has been transferred from New Haven to Camp Jackson, Columbia, S. C.

Major John A. Rafter of N. Tonawanda, formerly an editor of this journal, has been in charge of the establishment of emergency hospitals at training camps. He visited his home on leave in Nov.

To Camp Crane, Lieut. D. S. Dooman, Buffalo.

To Camp Dix, Lieut. R. E. Elliott, Rochester.

To Camp Dodge, Capt. Ross B. Nairn, Buffalo.

To Camp Holabird, Lieut. B. J. Slater, Rochester.

To Fort Oglethorpe, Lieut. A. Winkelstein, Syracuse.

To Fort Slocum, Lieut. F. N. Potts, Buffalo.

To New Haven, Lieuts. W. G. Hayward, Jamestown; W. E. Zielenski, Buffalo.

To report to the commanding officer, Eastern Department, Lieut. S. W. Thompson, Oswego.

The following have received orders, having already been in active service:

Capt. E. C. Koenig, Buffalo, to Camp Crane, Pa.

Lt. R. G. Fowler, Buffalo, to Camp Upton.

Lt. L. H. Wheeler, Lockport, to Ft. Oglethorpe for instruction.

The following have been commissioned and given first orders:

Lt. R. S. Cooper, Syracuse, to Camp Devens.

Major J. P. F. Burke, Buffalo, to Base Hospital, Camp Jackson, S. C., for instruction.

Capt. B. J. Bixby, Buffalo, to Ft. Oglethorpe for instruction.

Capt. T. D. Buck, Rochester, same.

Capt. T. F. Cole, Romulus, same.

Lt. H. H. LeSeur, Batavia, same.

Lt. H. M. Kenyon, Binghamton, same.

Lt. O. S. McKee, Buffalo, same.

Lt. A. L. Weil, Buffalo, same.

Lt. S. T. Hamilton, Elmira, same.

Lt. F. H. Goddard, Rochester, same.

Lt. A. C. Scinta, Rochester, same.

Lt. E. J. Wendel, Rochester, same.
Lt. A. T. Lawless, Syracuse, same.
Capt. C. J. Carr, Buffalo, to Hoboken.
Capt. E. A. French, Rochester, same.
Lt. R. Robinson, Lackawanna, same.
Lt. G. M. Brandt, Seneca Falls, to Camp A. A. Humphreys.
Capt. F. A. Mendlein, Buffalo, to Camp Devens.
Lt. F. Barber, Rochester, to Camp Holabird, Md.
Lt. A. Regan, Buffalo, to Camp Lee.
Lt. F. M. Carpenter, Rochester, to Camp Lee.
Capt. W. H. Mansperger, Buffalo, to Base Hospital, Camp Meade, for instruction.
Lt. J. F. Beiermeister, Rochester, same.
Lt. H. C. Schuhr, Buffalo, to Camp Sevier.
Lt. R. V. Laurence, Rochester, to Camp Sevier.
Capt. W. J. Bott, Buffalo, to Camp Upton.
Lt. E. J. Wendell, Rochester, to Camp Zachary Taylor.
Capt. E. H. Vail, Churchville, to Ft. Oglethorpe, for instruction.
Capt. E. J. Phillips, Corfu, same.
Capt. A. H. Rodgers, Corning, same.
Capt. J. J. Klein, E. Aurora, same.
Capt. H. C. Potter, E. Rochester, same.
Capt. A. M. Johnson, Rochester, same.
Capt. G. C. Whitney, Rochester, same.
Lt. O. E. White, Akron, same.
Lt. J. A. Kneller, Attica, same.
Lt. R. N. de Dominicis, Buffalo, same.
Lt. J. Heller, Buffalo, same.
Lt. A. W. Palmer, Buffalo, same.
Lt. M. A. Losee, Livingstonville, same.
Lt. J. C. Flynn, Rochester, same.
Lt. A. R. Hurley, Rochester, same.
Lt. R. B. Partridge, Rochester, same.
Lt. W. D. Wolff, Rochester, same.
Lt. D. G. Lenhart, Spencerport, same.
Capt. M. R. Waterman, Brockport, to Neurologic Institute, N. Y.
Capt. H. R. Lohnes, Buffalo, to Richmond, Va.
Lt. J. C. O'Gorman, Buffalo, to Camp Dix, Base Hospital.
Capt. D. S. Bellinger, Buffalo, to Base Hospital, Camp Meade.
Capt. R. S. Taylor, Buffalo, to Camp Shelby, Miss.
Capt. J. S. Lewis, Buffalo, to Ft. Oglethorpe, for instruction.
Capt. U. B. Stein, Buffalo, same.
Capt. Harry R. Trick, Buffalo, same.
Capt. F. Zingsheim, Buffalo, same.

Capt. B. A. Barney, Hornell, same.
Capt. F. R. Pickett, Olcott, same.
Lt. L. F. Anderson, Buffalo, same.
Lt. J. S. Gian Franceschi, Buffalo, same.
Lt. J. L. Kearney, Buffalo, same.
Lt. W. S. Lynch, Buffalo, same.
Lt. G. P. Michel, Buffalo, same.
Lt. F. Seilheimer, Buffalo, same.
Lt. F. Terrasse, Buffalo, same.
Lt. E. L. Villiaume, Buffalo, same.
Lt. C. A. Wisch, Niagara Falls, same.
Lt. R. P. Regan, N. Tonawanda, same.
Lt. C. J. Gian Franceschi, Rochester, same.
Lt. F. H. Stanbro, Springville, same.
Capt. E. G. Whipple, Rochester, to New Haven.
Capt. C. R. Orr, Buffalo, to Yale Army Medical School, for instruction.
Lt. L. J. Strong, Buffalo, same.
Lt. A. M. Wagner, Buffalo, to Camp Crane, Pa.
Capt. H. J. Bentz, Buffalo, to same for instruction.
Capt. J. H. Carr, Buffalo, same.
Lt. C. W. Bethune, Buffalo, to Camp Dix, Base Hospital.
Capt. A. E. Brennan, Buffalo, to Ft. Oglethorpe, for instruction.
Capt. F. St. John Hoffman, Buffalo, same.
Capt. F. B. Willard, Buffalo, same.
Lt. H. B. Brownell, Buffalo, same.
Lt. J. M. Flannery, Buffalo, same.
Lt. J. N. Kiefer, Buffalo, same.
Lt. S. A. Moore, Buffalo, to Ft. Oglethorpe, for instruction.
Lt. A. G. Ostwald, Buffalo, same.
Lt. F. H. Palmer, Buffalo, same.
Capt. G. A. Marion, Rochester, same.
Lt. P. C. Campbell, Buffalo, to Hoboken.
Lt. J. L. Gallagher, Buffalo, same.
Lt. E. P. Reimann, Buffalo, same.
Lt. J. A. Burns, Wilson, same.
Lt. F. D. Carr, Batavia, to Yale Army Laboratory School, for instruction.
Capt. G. C. Fisk, Buffalo, to N. Y. Neurologic Institute.
Capt. J. P. Barr, Buffalo, to commanding general, Southeastern Dept. (Charleston, S. C.)
Capt. B. H. Brady, Buffalo, same.
Lt. D. Brumberg, Buffalo, to Syracuse.
Capt. D. C. Main, Alfred, to St. Elizabeth's Hospital, Washington.
Capt. J. F. Shanahan, Buffalo, to same for intensive training.

Lt. V. Reinstein, Buffalo, same.

The following already in service have been ordered:

Capt. E. C. Koenig, Buffalo, to Cornell Medical College, N. Y.

Capt. H. B. Pritchard, Syracuse, to Camp Crane, Pa.

Lt. D. S. Dooman, Buffalo, same.

Lt. B. W. McCuen, Syracuse, to Camp Crane.

Lt. J. J. Finnegan, Buffalo, to Base Hospital, Camp Devens.

Lt. R. E. Elliott, Rochester, to Camp Dix.

Capt. B. Ross Nairn, Buffalo, to Camp Dodge, Ia., to examine the command for nervous diseases.

Lt. B. J. Slater, Rochester, to Camp Holabird, Md., (revoked).

Lt. A. Winkelstein, Syracuse, to Ft. Oglethorpe, for instruction.

Lt. F. N. Potts, Buffalo, to Ft. Slocum and on completion of duty to Washington. Later ordered to Camp Meade.

Lt. W. E. Zielenski, Buffalo, to New Haven.

Lt. F. C. McClellan, Canandaigua, same.

Lt. D. Jung, Buffalo, same.

Capt. J. R. Brownell, Perry, to Camp Crane, Pa.

Lt. R. L. Cooley, Buffalo, same.

Lt. M. A. Thompson, Buffalo, same.

Major H. R. Gaylord, Buffalo, to Camp Wheeler, Ga.

Major E. A. Southall, Buffalo, to Columbus, Ga.

Lt. P. H. J. Buckley, Buffalo, to Evacuation Hospital, Ft. Oglethorpe.

Capt. M. B. Palmer, Rochester, to Washington.

Lt. T. M. Calladine, Perry, to Army Medical School, for instruction.

Lt. J. F. Browne, Rochester, to Carnegie Institute and University of Pittsburgh.

Lt. J. J. Leary, Utica, to Camp Wadsworth, Va.

Lt. W. A. Behan, Binghamton, to Colonia, N. J.

Lt. H. L. Prince, Rochester, to Walter Reed Hospital, D. C.

Lt. T. L. McNamara, Corning, to Rahway, N. J.

Lt. J. L. Kinner, Elmira, to Rockefeller Institute, for instruction and then to Camp Meade, Base Hospital.

